# Advances in remote sensing of emperor penguins: first multi-year time series documenting trends in the global population

**DOI:** 10.1098/rspb.2023.2067

**Published:** 2024-03-13

**Authors:** Michelle LaRue, David Iles, Sara Labrousse, Peter Fretwell, David Ortega, Eileen Devane, Isabella Horstmann, Lise Viollat, Rose Foster-Dyer, Céline Le Bohec, Daniel Zitterbart, Aymeric Houstin, Sebastian Richter, Alexander Winterl, Barbara Wienecke, Leo Salas, Monique Nixon, Christophe Barbraud, Gerald Kooyman, Paul Ponganis, David Ainley, Philip Trathan, Stephanie Jenouvrier

**Affiliations:** ^1^ Department of Earth and Environmental Science, University of Minnesota, Minneapolis, MN, USA; ^2^ School of Earth and Environment, University of Canterbury, Christchurch, New Zealand; ^3^ Canadian Wildlife Service, Environment and Climate Change Canada, Ottawa, Canada; ^4^ Woods Hole Oceanographic Institution, Woods Hole, MA, USA; ^5^ Sorbonne Université, LOCEAN-IPSL, UMR 7159, 75005, Paris, France; ^6^ British Antarctic Survey, Cambridge, UK; ^7^ Centre National de la Recherche Scientifique, Université de Strasbourg, IPHC UMR 7178, Strasbourg, France; ^8^ Département de Biologie Polaire, Centre Scientifique de Monaco, Monaco City, Monaco; ^9^ Department of Physics, Friedrich-Alexander University Erlangen-Nürnberg, Erlangen, Germany; ^10^ Department of Climate Change, Energy, the Environment and Water, Australian Antarctic Division, Hobart, Australia; ^11^ Point Blue Conservation Science, Petaluma, CA, USA; ^12^ Centre d'Etudes Biologiques de Chizé, UMR7372 Centre National de la Recherche Scientifique – La Rochelle Université, 79360 Villiers en Bois, France; ^13^ Scripps Institution of Oceanography, La Jolla, CA, USA; ^14^ HT Harvey and Associated, Los Gatos, CA, USA; ^15^ Ocean and Earth Science, National Oceanography Centre, University of Southampton, University Road, Southampton SO17 1BJ, UK

**Keywords:** Antarctica, high-resolution satellite imagery, Bayesian modelling, Southern Ocean, *Aptenodytes forsteri*

## Abstract

Like many polar animals, emperor penguin populations are challenging to monitor because of the species' life history and remoteness. Consequently, it has been difficult to establish its global status, a subject important to resolve as polar environments change. To advance our understanding of emperor penguins, we combined remote sensing, validation surveys and using Bayesian modelling, we estimated a comprehensive population trajectory over a recent 10-year period, encompassing the entirety of the species’ range. Reported as indices of abundance, our study indicates with 81% probability that there were fewer adult emperor penguins in 2018 than in 2009, with a posterior median decrease of 9.6% (95% credible interval (CI) −26.4% to +9.4%). The global population trend was −1.3% per year over this period (95% CI = −3.3% to +1.0%) and declines probably occurred in four of eight fast ice regions, irrespective of habitat conditions. Thus far, explanations have yet to be identified regarding trends, especially as we observed an apparent population uptick toward the end of time series. Our work potentially establishes a framework for monitoring other Antarctic coastal species detectable by satellite, while promoting a need for research to better understand factors driving biotic changes in the Southern Ocean ecosystem.

## 1. Introduction

Emperor penguins (*Aptenodytes forsteri*) form breeding colonies on seasonal sea ice fastened to land (‘fast ice’ [1]) during winter, when Antarctic research is especially difficult—it is dark and cold. Thus, more so than other wildlife in the Southern Ocean, emperor penguin populations have only been monitored at the very few locations close to research bases where direct counts are possible. For example, the only mark–recapture, demographic study on emperor penguins conducted thus far was at Dumont d'Urville Station (1962–1988; 2010–present), representing the longest continuous study involving more than counting and biologging [2]. The demographic study found a substantial drop in breeding pairs in the 1970s followed by marked stability at a population level half its previous size (though the population has increased again recently). Whether or not part of this decline, in addition to increased mortality of adult males [2], represented emigration to a nearby colony (detectable in recent satellite imagery) is unknown. In contrast, counts, but not demographic analysis, at Cape Crozier in the Ross Sea over a period of approximately 60 years indicate colony size fluctuations [3,4]. Effective understanding of colony dynamics, thus, requires a regional perspective [5]. Recently, with the development of high-resolution satellite imagery (VHR; 30–60 cm spatial resolution), detecting and quantifying populations of many animal species (e.g. wildebeest (*Connochaetes taurinus*), polar bears (*Ursus maritimus*) and cattle (*Bos taurus*) [6–8]) has become possible, including ice-obligate, Antarctic species (Adélie penguins (*Pygoscelis adeliae*); Weddell seals (*Leptonychotes weddellii*) [9–11]). The same is true for emperor penguin colonies, which owing to the dark–light contrast of guano on sea ice, are easily detectable on images of ice [12,13]: using 2009 VHR images, 46 emperor penguin colonies were found around the Antarctic coastline, and authors reported a first baseline population estimate of approximately 238 000 breeding pairs [12]. Several colony locations have since been added, bringing the total to more than 60 known locations where emperor penguins have been observed to form a colony at least once [14,15]. It is now highly probable that most emperor penguin colony locations have been detected [15,16], about half via satellite imagery. The technology thus has proved to be invaluable for consistently monitoring populations of wildlife, especially in remote locations such as most of coastal Antarctica.

Here, with the satellite record increasing in length, we assess 10 years of changes in emperor penguin colonies across multiple spatial scales (colony, regional and global). This assessment is critical for better understanding emperor penguins' fate as sea ice responds to climate change [17] and industrial fishing continues (e.g. for Antarctic krill, *Euphausia superba*; Antarctic toothfish, *Dissostichus mawsoni*) [18]. We analysed 460 VHR images [12–16,19–21], aerial surveys across several colonies [22–24], ground surveys [25] and remote camera data [26]. Remote sensing analysis involved a semi-automated, supervised classification of VHR images combined with aerial and ground validation to convert the area covered by ‘penguin pixels’ into indices of population size (number of adult birds present in colonies during aerial surveys) [12,19]. We integrated available survey data in a Bayesian state-space population model, allowing us to estimate annual population indices and trends from 50 colony locations (that were discovered prior to 2015) during the period 2009–2018. From these results, we looked to see whether any trends were evident. We hypothesized that changes in population indices over 10 years would vary regionally, being positively correlated with sea ice conditions. Based on previous modelling work [27], we hypothesized that global population indices would be smaller in 2018 than in 2009. Our aim was to report these estimates (indices) as open data, and summarize population trends regionally, to quantify Southern Ocean environmental trends in relation to fast ice [28] and pack ice [29].

## 2. Material and methods

Our study area included the entirety of the fast ice available around the Antarctic coastline (emperor penguin habitat) during spring (August–November) 2009–2018 and focused on all known locations of emperor penguin colonies [12,15] that were discovered prior to 2015, and when we knew chicks would be present. Thus, some colonies could well have started but then failed entirely. Locations for emperor penguin colonies are routinely updated as new colonies are discovered [20,21] (and many recent additions have been quite small) but our initial acquisition began with the initial 46 colonies reported in the first global census [12]; but we added four others to total 50. Our primary objective was to use VHR satellite imagery to learn about the status and trends of the global population during a recent 10-year period. Aerial surveys, ground and remote camera data reported in the Mapping Application for Penguin Populations and Projected Dynamics (MAPPPD [4]) were also used as ground truthing of imagery.

### (a) Aerial surveys

During the 2018 Antarctic field season, under permit no. 2019-006 granted by the National Science Foundation, our US-based team conducted aerial photography at emperor penguin colonies in the Ross Sea to add to robust validation of imagery. Our efforts included one flight via fixed wing aircraft over colonies distant from McMurdo Station and five flights via helicopter to a single colony (Cape Crozier) near the station. The five flights to Cape Crozier, 24 October to 15 November, were used to better understand population fluctuation through a single season. Our fixed wing survey took place on 31 October 2018, flying in the vicinity of Beaufort Island (Antarctic Specially Protected Area (ASPA) 105), Franklin Island, Cape Washington (ASPA 173), Coulman Island and Cape Roget. At each location (both by fixed wing and helicopter), we circled the colony one–four times, maintaining a minimum of 500 m horizontal distance from the periphery of the colony and a minimum altitude of 500 m [30]. No behavioural disturbance to birds (e.g. rapid movement or dispersion of adults or chicks) were noted during any flight. Oblique photographs (with a Canon EOS Mark 7D II with Tamron 400 m zoom lens) were taken through the window of the Basler aircraft, and in the case of our AStar helicopter surveys with the window down, in continuous shooting mode to ensure effective ability to stitch photos together for counting.

We then downloaded and stitched the multiple photos per colony with Adobe photo-stitching software to create a single image for manual counting. We loaded images of colonies into the free software ImageJ [31], which allowed us to document and assess precision of counts among observers. Our field team (four people) counted the largest colony in the world, Coulman Island [12], to gain an understanding of among-counter precision. After determining a coefficient of variation of approximately 2.5% (small variation in counts among observers), we determined that each team member would be assigned to count one of the remaining colonies each, to speed the process and to arrive at a population count of adult emperor penguins at each of six Ross Sea colonies during spring 2018. These data were immediately entered into MAPPPD repository [4]. We used these counts as validation for our observation model (see population modelling below).

### (b) Satellite imagery

To gather images for analysis, we first reviewed discover.digitalglobe.com (Maxar Technologies) to determine image availability per emperor penguin location per year, and to determine utility/quality of images for analysis [12,32]. Images had been requested for acquisition via the National Geospatial Intelligence Agency (NGA), when possible, once per month during cloud-free days in austral spring. We avoided images with excessive clouds, and those that were too dark, too bright, or otherwise low quality. We created a list comprising the unique identifier for each image (called the ‘catalog ID’) and then requested images be processed (via Polar Geospatial Center (PGC)), specifically pan-sharpened (i.e. increasing the spatial resolution of the multi-spectral image by merging it with its higher-resolution, panchromatic pair [33]) and projected to Antarctic Polar Stereographic (EPSG code 3031). We then followed semi-automated methods already established [12,13,19]: briefly, we loaded VHR imagery into ArcGIS 10.8 (Esri), identified the location of emperor penguins on the image and then clipped images to the extent of the colony. We conducted a supervised classification by manually training the program with shapefile points representing pixels of guano, snow and penguin. We conducted a maximum-likelihood classification based on these classes to arrive at a classified raster image identifying pixels that are probably penguin pixels. Our final step was to convert the raster to a polygon shapefile and to calculate the area (m^2^) of penguin pixels. The area of ‘penguin pixels’ per colony per year served as the response variable and input to the population modelling (below). We conducted this process for all 50 colonies in all years for which imagery existed during 2009–2018.

### (c) Model overview

We developed a Bayesian state-space model to accommodate several key features of emperor penguin population dynamics and the data collection (i.e. observation) process. We gathered all available adult count data (obtained from remote cameras, ground and aerial surveys) from MAPPPD [4] for colonies that ranged in size and in proximity to research stations, and which were situated in different regions of the Antarctic.

The population processes we sought to model were:

(1) colony-level trends and annual fluctuations can differ, even among nearby colonies;
(2) individual colonies can temporarily ‘vanish’ for a breeding season and reappear in future years (somewhat depending on fast ice conditions);
(3) daily abundance of adult penguins in a colony can vary substantially throughout the spring survey period, caused by breeding synchrony, temporal variation in adult foraging trips, the presence/absence of non-breeding adults, emigration from the colony, breeding failure and changes in parenting behaviour (crèching). As noted, unknown to us was the prevalence of these factors before images were acquired, thus subsequently affecting what we measured as ‘colony size’.

The data collection (i.e. observation) processes we sought to model were:

(4) counts of adults from aerial surveys are an imperfect observation of the seasonal population index (i.e. due to counting errors during surveys and intraseasonal variation in daily abundance of adults at colonies; point 3 above);
(5) satellite observations of the ‘area of ground occupied by penguins' are imprecise and potentially biased estimates of the true count during the survey, and by extension, of the seasonal expected count;
(6) the expected number of birds counted at a colony (either through aerial surveys or satellite images) potentially changes over the survey period, owing to chick mortality and subsequent emigration by attendant adults.

The equations describing the model are detailed in electronic supplementary material, tables S1 and S2, and more fully explained below.

### (d) Population process model

The population model describes the expected abundance at colony *j* in year *y* as a mixture of an abundance term and a colony presence term $z_{\;j,y}$,

$$N_{\;j,y}=\;z_{\;j,y}X_{\;j,y}.$$

We model $X_{\;j,y}$ a stochastic process controlled by a colony-specific parameter $r_{j}$ that measures the expected annual difference between years and a lognormal variance term $\sigma_{\mathrm{process}}^{2}$,

$$X_{\;j,y}∼\mathrm{lognormal}(\log(X_{\;j,y-1})+r_{j},\sigma_{\mathrm{process}}^{2}).$$

Colonies are occasionally apparently absent in certain years, owing to factors such as early sea ice breakup. We model this as a Bernoulli process controlled by a probability parameter p,

$$z_{\;j,y}∼\;\mathrm{Bernoulli}(p).$$

In our 10-year time series, there were too few instances of apparent colony absences to model *p* as a colony-specific parameter, or to evaluate if the probability of colony presence depended on abundance in previous years. So, though the occupancy model aims to address the probability of penguins showing at a location, it lacks a detectability component where individuals may still be present but were missed. However, we recommend addressing detectability as a priority for future work as more monitoring data are collected.

We model $r_{j}$ as a colony-level random effect arising from a globally shared distribution,

$$r_{j}∼\mathrm{Normal}(\bar{r},\sigma_{r}^{2}).$$

This random effect parametrization regularizes estimates of annual change for colonies with sparse data and ensures that spatial variance in $r_{j}$ is not overestimated, which could erroneously inflate estimates of global population growth rates [34].

The model requires a prior for $X_{\;j,1}$ at each colony, analogous to an intercept term in linear regression. We assumed the values of $X_{\;j,1}$ were lognormal random variables drawn from shared global distribution of colony abundances with hyperparameters estimated empirically from the data, thereby constraining estimates of initial abundances for colonies with extremely sparse data to a range that is consistent with those observed at other colonies. This was accomplished using

$$X_{\;j,1}∼\mathrm{lognormal}(\log({\bar{X}}_{1}),\sigma_{\log X_{1}}^{2}).$$

### (e) Aerial observation model

Because abundance of adults within colonies fluctuates day to day over the survey period, even a highly precise count from a single aerial photograph is not necessarily an accurate estimate of $N_{\;j,y}$ (the seasonal mean count, which is our index of abundance). Additionally, repeated observations at several colonies suggest that colony abundance of breeding adults declines over the course of the spring survey period as birds depart the colony following reproductive failure. Non-breeders probably also gradually depart. Our aerial observation model therefore includes log-linear fixed effect (α) to account for the day of the year (DOY) on which surveys were collected. For each observation, we calculated the DOY covariate by subtracting 289 (the mid-point of the survey period) from the date of the survey. The model also includes a variance term ($\sigma_{\mathrm{aerial}\_\mathrm{obs}}^{2}$) that encapsulates both counting errors during aerial surveys and residual day-to-day variance in the numbers of adults present in the colony during the survey period. Accordingly, the expected aerial count on day *i* ($\lambda_{\;j,y,i}),$ conditional on colony presence ($z_{\;j,y}=1$), was modelled as

$$\lambda_{\;j,y,i}∼\mathrm{lognormal}\left(\log(X_{\;j,y})+\alpha \times {\mathrm{DOY}}_{\;j,y,i}\;-\frac{1}{2}\sigma_{\mathrm{aerial}\_\mathrm{obs}}^{2},\sigma_{\mathrm{aerial}\_\mathrm{obs}}^{2}\right).$$

The term $-\frac{1}{2}\sigma_{\mathrm{aerial}\_\mathrm{obs}}^{2}$ is a lognormal variance correction that ensures $\lambda_{\;j,y,i}$ is centred on the mean of $X_{\;j,y}$ rather than the median. In other words, it is a correction term that ensures the sampled values resemble the observations by removing the confounding effect of sampling error and daily variance in penguin attendance. There were not enough repeated observations to model α separately for each colony, but we note this is an important future direction given that seasonal phenology probably differs among colonies. Since α is fit on a log-linear scale, it describes the proportional change in expected count for each day elapsed in the survey season.

### (f) Satellite observation model

Our satellite observation model assumes that estimates of area occupied by penguins in satellite images are normally distributed and centred on the expected number of penguins present in the colony on the day of the survey, which is equal to $N_{\;j,y}\exp(\alpha \times {\mathrm{DOY}}_{\;j,y,i})$, multiplied by a constant that accommodates bias resulting from interpretation of satellite images (β). This is equivalent to assuming satellite observations have a constant proportional bias. As with aerial survey dates, we calculated the DOY covariate for each satellite image by subtracting 289. Rather than assuming homoscedastic variance in satellite observations, we assumed a constant coefficient of variance in satellite observations such that $\;\omega_{\;j,y}^{2}=\;{\left(N_{\;j,y}\exp(\alpha \times {\mathrm{DOY}}_{\;j,y,i})\times {\mathrm{CV}}_{\mathrm{satellite}}\right)}^{2}$, which also ensures that $\;\omega_{\;j,y}^{2}$ is zero (no error) when a colony is truly absent in a year. Satellite ‘counts’ were therefore linked to colony abundance through

$$S_{\;j,y,i}∼\mathrm{normal}(N_{\;j,y}\exp(\alpha \times {\mathrm{DOY}}_{\;j,y,i}),\omega_{\;j,y}^{2}).$$

This implies that satellite observations at larger colonies have larger absolute error but equivalent proportional error compared with smaller colonies. Visual inspection of satellite data further confirmed this was an appropriate assumption.

Each satellite image was assigned a subjective ‘quality’ score (1 = poor, 2 = moderate, 3 = good). We estimated separate fixed effects for β and ${\mathrm{CV}}_{\mathrm{satellite}}$ for each level of image quality, to allow for different biases and precision associated with different image quality. As illustrated in electronic supplementary material, table S3, poor-quality images had more bias (β farther from 1) and higher values of ${\mathrm{CV}}_{\mathrm{satellite}}$ than good-quality images.

### (g) Model fitting

All data were analysed using R version 4.2 [35], with posterior samples generated using Markov chain Monte Carlo methods implemented using JAGS version 4.3. After a burn-in period of 50 000 iterations, we stored every 50th iteration until we accumulated 10 000 posterior samples from each of three Markov chains. The model unambiguously converged; the Gelman–Rubin convergence statistic was less than 1.1 for all hyperparameters, colony- and year-level effects, regression coefficients, and latent states. We confirmed that the effective sample size for each parameter was greater than 2000 and also confirmed the ability of the model to generate data that are consistent with the observed data, using posterior predictive checks. We confirmed the ability of our model to generate unbiased and identifiable trend estimates using simulations. We report the medians and 95% equal-tailed credible intervals (CIs) of all modelled quantities unless otherwise noted.

### (h) Goodness-of-fit and model diagnostics

Posterior predictive checks confirmed that the fitted model could generate data with reasonable properties and no obvious systematic discrepancies with the observed data (i.e. data simulated from the fitted model ‘looks like’ the observed data). Of our simulated datasets, 31% had aerial observations with lower root mean square error (RMSE) than the observed data (i.e. Bayesian *p*-value = 0.31) and 29% of simulated datasets had satellite observations with lower RMSE than observed data (i.e. Bayesian *p*-value = 0.29; electronic supplementary material, figure S1). Bayesian *p*-values close to 0.5 indicate a reasonable fit and occur when the fitted statistical model is equivalent to the ‘true’ model that generated the data. Visual inspection of observed versus fitted values (electronic supplementary material, figure S2) also indicated the model was a good fit to the data.

### (i) Simulations to confirm parameter identifiability

We conducted a series of simulations (*n* = 1000) to confirm that the statistical model could generate identifiable and unbiased estimates of global population trend and change, given realistic data availability, observation error and survey imbalance among colonies. In each simulation, we generated a time series of ‘true’ abundance at each of the 50 colonies (also resulting in a simulated global trend), and then simulated aerial and satellite observations at each colony, including realistic data imbalance, as well as aerial and satellite observation error (based on values estimated from the analysis of empirical data; electronic supplementary material, table S3). We then used these simulated observations as ‘data,’ refit our statistical model to those simulated data, and evaluated whether we could recover unbiased estimates of global trend with appropriate CI coverage (electronic supplementary material, figures S3 and S4). Simulations indicated that the model could reliably recover estimates of global trend (median bias = 0.3%; electronic supplementary material, figure S3) and change between 2009 and 2018 (median bias = 3.5%; electronic supplementary material, figure S4), with appropriate 95% CI coverage. These simulations suggest that the extreme data imbalance in our data, coupled with our choice of priors, does not induce severe bias into estimates of population change.

### (j) Sea ice correlations

To investigate a possible relationship between regional trends in fast ice or pack ice, we gathered published data on fast ice trends [28] and pack ice trends [29] within discrete regions of Antarctica that differ in their patterns of ice formation and sea ice co-variability. We then assigned each emperor penguin colony to these sea ice regions. Within each region, we used samples from the Bayesian joint posterior to sum colony indices and thereby calculate estimates of regional indices and population change. Finally, we estimated the Spearman rank correlation between regional population and regional trends for fast ice (*n* = 8), and pack ice (*n* = 5).

## 3. Results and discussion

**Figure 1.**
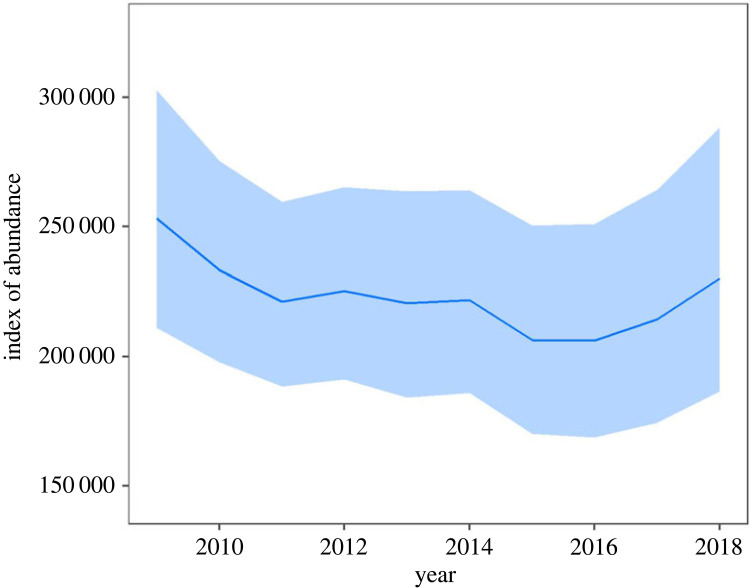
Index of global abundance of emperor penguins (number of adult birds in attendance at colonies in the springtime [19]) per year calculated by combining area of ‘penguin pixels’ from VHR imagery within a Bayesian modelling framework over the study period (2009–2018). The blue ribbon represents the 95% equal-tailed CI for the annual index, while the central line represents the median of the posterior distribution.

We found an 81% chance that the global emperor penguin population was smaller in 2018 than 2009, representing a probable 9.6% decrease in the index of global abundance (95% CI = −26.4% to +9.4%); or approximately 24 000 fewer adults in attendance at breeding colonies during spring (approximately 252 000 decreasing to approximately 228 000; figure 1). The global population trajectory over this period, measured as the log-linear annual rate of change, was −1.3% per year (−3.3% to +1.0%). However, the overall population decreased for the first few years, but then exhibited an increasing trend (though not reaching the initial level; figure 1). Our analysis also updates a previously estimated global abundance in 2009 [12] from approximately 576 000 breeding adults (approximately 238 000 breeding pairs) to a new index of abundance of approximately 252 000 breeding adults, by altering an assumption about what proportion of the population is detected. Rather than one individual equalling one pair, both members are often present at any one time regardless of census method. We also note that our estimate includes only the proportion of the population present in late spring (and is therefore a conservative index of adult emperor penguins), which includes an unknown quantity of failed-breeders, pre-breeders and non-breeders.

**Figure 2.**
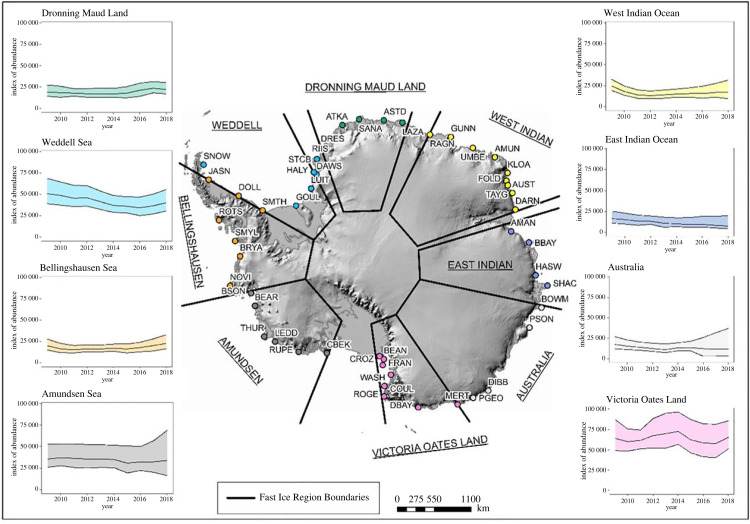
Regional dynamics of emperor penguin populations (indices of abundance of adult penguins observed from VHR imagery during each austral spring on the *Y*-axis, which is fixed from 0 at the origin to a maximum of 100 000 birds; colony codes defined in [5]), defined by fast ice region [28] during the study period (2009–2018). For inset charts, the central lines represent the median of the posterior distributions, while outer bands represent 95% equal-tailed CIs for the annual indices.

**Figure 3.**
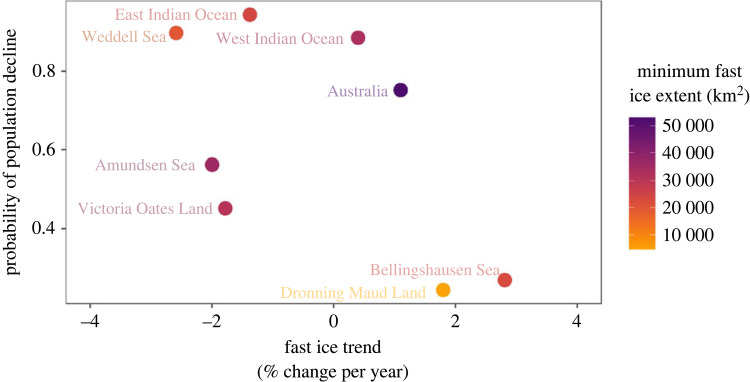
Relationship between probability of regional population decline of emperor penguins (indices of abundance of adult birds present in springtime at colonies during 2009–2018) and 18-year trend in regional fast ice extent (from table 1 in [28]). Dots are coloured by the mean annual minimum fast ice extent across the 18-year period to differentiate regions with low persistent sea ice (e.g. Dronning Maud Land) from regions with abundant annual sea ice (e.g. Australia).

Emperor penguins form colonies and rear their chicks primarily on fast ice (or low-lying ice shelves [1,12,20]). Researchers recently partitioned fast ice into eight regions based on decades-long trends in extent [28]; trends in our indices of emperor penguin populations differed among these regions over the 10 years. In half of the eight fast ice regions penguin numbers declined, though drastic decline (greater than 50%) was unlikely (figure 2 and table 1). The greatest probability of declines in regional populations were in the East Indian Ocean and the Weddell Sea sectors, where fast ice extent (i.e. the distance from the shore to the edge of the fast ice) has generally decreased in recent decades [28]. By contrast, populations appear to have remained stable or shown an increase in Dronning Maud Land and the Bellingshausen Sea sectors where fast ice extent increased during our study period (table 1). Across all eight regions, trends in fast ice extent were only weakly correlated with the probability of population declines (figure 3; Spearman rank correlation = −0.52), suggesting that other factors must be at play.

**Table 1.** Measures of population change across 50 emperor penguin colonies during 2009–2018, including mean and standard error (s.e.), based on grouping colonies within fast ice region (top) and pack ice region (bottom).

	*n* (colonies)	probability of decline (%)	probability of 30% decline (%)	probability of 50% decline (%)	mean change (%)	s.e.
fast ice region						
Amundsen Sea	6	56.1	20.0	5.1	2.2	41.9
Australia	4	75.0	51.3	27.7	−18.1	54.7
Bellingshausen Sea	7	27.3	1.1	0.0	16.2	25.2
Dronning Maud Land	5	24.4	1.0	0.0	17.8	24.7
East Indian Ocean	4	94.3	82.2	57.3	−46.2	31.0
Victoria Oates Land	8	45.5	1.9	0.0	3.5	18.5
Weddell Sea	7	89.5	24.2	0.6	−19.1	15.1
West Indian Ocean	9	88.6	51.5	14.1	−27.3	23.8
pack ice region						
Bell-Amundsen	8	51.8	3.9	0.0	2.1	22.6
Indian	11	92.7	55.4	12.5	−29.7	19.9
Pacific	8	68.8	24.2	2.6	−7.8	32.9
Ross	8	56.7	7.3	0.2	−0.6	23.6
Weddell	15	79.7	3.3	0.0	−9.6	12.1

**Figure 4.**
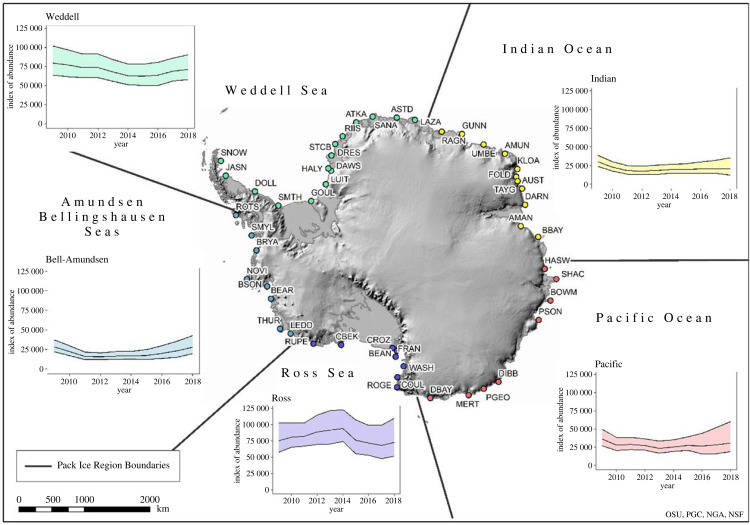
Regional dynamics of emperor penguin populations (indices of abundance of adult penguins observed from VHR imagery during each austral spring on the *Y*-axis, which is fixed from 0 at the origin to a maximum of 125 000 birds; colony codes defined in [5]), defined by pack ice region [29] during the study period (2009–2018). For inset charts, the central lines represent the median of the posterior distributions, while outer bands represent 95% equal-tailed CIs for the annual indices.

Emperor penguins are reliant on pack ice as their main prey are generally associated with sea ice [36–40], and during moult the birds need to stand for a few weeks on a stable, wind-protected surface [41,42]. As such, we also evaluated population trends within five broad Antarctic pack ice regions [29]. We suggest abundance indices probably declined in most pack ice regions, though population trajectories differed within these regions as well (table 1). For example, populations in the Weddell Sea sector declined on average by approximately 10% over the study period, largely driven by losses at Halley Bay (where the index dropped from approximately 20 000 birds in 2009 to less than 1000 by 2018), while indices in the Indian Ocean sector declined by 30% (table 1). In the Bellingshausen–Amundsen sector, where an Antarctic-wide, climatic regime shift resulted in particularly variable sea ice extent [43] there, populations experienced a steep decline from approximately 29 000 individuals in 2009 to approximately 16 000 in 2012—but with a substantial uptick in population from 2016 to 2018 (figure 4). The latter is interesting, given the steep decrease in overall Southern Ocean pack ice area during 2014–2018, returning (at least in the satellite era) to what it had been before a decades-long gradual increase. Analysis of what the multi-scale feedbacks might be between ice extent and population size has yet to be well investigated.

Like other species using an ephemeral habitat, temporal dynamics at individual emperor penguin colonies can be highly variable. Annual indices frequently changed by more than 25% between consecutive years within a colony, and one well-surveyed colony (Point Géologie, near Dumont d'Urville station) experienced a decline of approximately 50% from 2012 to 2013, followed by an increase of approximately 100% 2 years later (electronic supplementary material, figure S2). The same sort of dynamic is evident for colonies in the Ross Sea [22,23]. Additionally, eight colonies apparently failed to form in at least one year during the study period, though adults were detected at these colonies in subsequent years (a phenomenon previously referred to as ‘colony blinking’ [14,44]; electronic supplementary material, table S4). High temporal variance (i.e. process variation) at colonies has been reported previously [22,23,25,45,46] and is expected because fast ice can be ephemeral and thus the species has evolved a life history that prioritizes adult survival over reproduction, especially in difficult conditions. Though we tried to account for detectability in the occupancy for each site, we acknowledge that other components were not included, such as the effect of temporary migrations that would violate the closure assumption of the detection model. Variance in annual counts may also be driven by movement of individuals between adjacent colonies among years [14,23]. A notable example of such movement occurred at Dawson–Lambton, which appears to have recruited many thousand individuals from nearby (and declining) Halley Bay [45]. Importantly, there are now 66 emperor colonies on record [47], most of which were discovered for the first time after 2009 [15]—thus, it is possible that movement away from the 50 colonies in our study partly explains the decrease we observed over 2009–2018. As a consequence, at the colony scale many years of monitoring will typically be required to generate precise estimates of trends; and even precise estimates of change at a single colony may fail to generalize to other locations, as has been shown with the much more well-studied Adélie penguin [48]. Multi-scale surveys will continue to be important, because notably the biggest contributors to the global trend came from two regions: the Ross and Weddell Seas, highlighting the potential for our work to inform future conservation policy. Thus, any robust signal of global population change would only be apparent when aggregating information across the species range, as reported here.

Understanding the demographic drivers of population change remains a critical information gap for emperor penguins, as with many long-lived species. The declines we detected could be consistent with altered breeding propensity [49] (including after major perturbations that may extend across a number of years, such as reported for Halley Bay [45]), increased reproductive failure and/or increased mortality of any age class, especially lower recruitment [50,51]. Each of these mechanisms could have different effects on population structure and viability that cannot be determined with colony surveys alone [52]. Seabird populations also include substantial ‘unobservable’ life history stages (pre-breeding juveniles, non-breeding adults) that strongly affect population viability but are difficult to monitor directly because these stage classes are absent from colonies at the time of the count. For example, demographic monitoring of Tristan albatross (*Diomedea dabbenena*) revealed that reproductive failure diminished the abundance of unobservable (non-breeding and pre-breeding) stage classes across an 18-year period, despite apparent stability in the number of observable breeders [53]. Because we are similarly capturing a subset of the population (and in our case, not the breeding population), the framework we present here may represent an index of demographic parameters for emperor penguins, such as breeding success, of which sustained changes would ultimately influence the unobservable breeding population of emperor penguins (during austral winter).

Previously, model projections have predicted declines in the emperor penguin population through to the end of this century [27,54,55], but observations have been missing until now. In the absence of any estimates for emperor penguin vital rates, beyond those from one well-studied colony [2,56,57], metapopulation modelling remains challenging. With the results from our study, there is now the potential to make more informed predictions combining monitoring methods and technologies (e.g. field-based and remote sensing), although these will require evaluation of assumptions regarding observation and process errors associated with models.

Our work documents population change in an iconic polar seabird and demonstrates the utility of using satellite imagery to empirically enumerate its and other wildlife populations remotely. In a rapidly changing world, new approaches will be necessary if we are to better understand the consequences of climate change and the consequences for species, especially in remote locations that are challenging to access. Remote sensing observations, critically, need to be complemented by field-based methods (e.g. observational, biologging and/or molecular methods [22–24,58–62] and long-term (i.e. multiple generations) data to contextualize short-term trajectories, and to understand demographic and ecological mechanisms that explain trends in population indices. For emperor penguins, such methods now have the potential to help with assessing population status and trend at a global scale, providing an invaluable tool for adaptive conservation planning in a changing Southern Ocean.

## Data Availability

Data, metadata and code required to replicate our results are provided open-source via Dryad [63]: https://doi.org/10.5061/dryad.m63xsj48v. This repository contains: (1) all area estimates derived from remote sensing analysis; (2) aerial survey and ground count estimates of emperor penguins at all colonies for which data are available; (3) all code to prepare and run the Bayesian model. High-resolution satellite images are copyright Maxar Technologies (formerly, DigitalGlobe, Inc), were obtained via the United States National Geospatial Intelligence Agency (NGA), and were processed for use and distributed by the Polar Geospatial Center (University of Minnesota) through the Nextview license. The unique identifiers for each image are available within the ‘area estimates’ dataset such that independent acquisition and analysis of the images can be conducted to replicate the area estimates derived from satellite imagery. Code used to process satellite imagery from raw images to pan-sharpened images can be found here: https://www.pgc.umn.edu/guides/pgc-coding-and-utilities/using-pgc-github-pansharpening/. All aerial and ground-based estimates of emperor penguin populations were obtained from the open-source Mapping Application for Penguin Populations and Projected Dynamics (penguinmap.com). Code for running the Bayesian model can be found here with no restrictions: https://doi.org/10.5061/dryad.m63xsj48v [63]. Code required to process high-resolution satellite imagery can be found here with no restrictions: https://www.pgc.umn.edu/guides/pgc-coding-and-utilities/using-pgc-github-pansharpening/. The data are provided in electronic supplementary material [64].
